# Effect of online intervention mode on breastfeeding results: a systematic review and meta-analysis

**DOI:** 10.1186/s12978-023-01701-0

**Published:** 2023-11-06

**Authors:** Yue Sun, Yutong Gao, Zhiling Zhu, Lili Zhu

**Affiliations:** 1 Department of Nursing, School of Nursing, Xinxiang Medical University, Xinxiang, Henan China; 2 Xinxiang Central Hospital, Xinxiang, Henan China

**Keywords:** Breastfeeding, Internet, Intervention, Meta-analysis

## Abstract

**Objective:**

To evaluate the effect of Internet based intervention model on breastfeeding knowledge, attitude, self-confidence and breastfeeding rate.

**Methods:**

An electronic literature search of PubMed, Embase, Web of Science, Cochrane Library, CINAHL, CNKI, Wanfang, CBM and VIP database was performed using combinations of the keywords “Breastfeeding,” “Breastfeeding, Exclusive,” “Internet,” “Online,” and “Website”. The retrieval period was from their inception to March 31, 2023. Quality appraisal was performed using the Cochrane 5.1 for randomized controlled trials (RCTs). RevMan5.3 was used for data analysis.

**Results:**

Thirty-two studies were included in the review, with a total of 9514 samples. The results of Meta-analysis showed that, compared with routine nursing, the intervention model based on the Internet can effectively improve breastfeeding knowledge and attitude of pregnant women, improve breastfeeding self-confidence (*P* < 0.05), and improve the rate of exclusive breastfeeding in the short term (within 6 weeks) and the long-term postpartum (3–6 months) had a positive effect on the rate of exclusive breastfeeding (*P* < 0.05).

**Conclusions:**

The Internet breastfeeding intervention model may be an effective intervention to improve the effect of exclusive breastfeeding. In the future, more high-quality, large-sample randomized controlled trials can be carried out to further explore the effect of the Internet intervention model on breastfeeding.

**Supplementary Information:**

The online version contains supplementary material available at 10.1186/s12978-023-01701-0.

## Introduction

Breastfeeding is an ideal way to provide infants with nutrition needed for healthy growth and development. Breastmilk contains rich nutrients, growth regulators, anti-inflammatory factors and other immune active substances beneficial to children's growth and development, which can meet all the nutrients required for the growth and development of infants aged 0–6 months [1]. The World Health Organization (WHO) recommends that infants should be exclusively breastfed within 6 months of birth, and breastfeeding can continue until the infant is 2 years old or above [2]. However, according to data released by the China Development Research Foundation in 2019, the rate of exclusive breastfeeding for infants within 6 months in my country is only 29%, far below the target of 50% of infants aged 0 to 6 months set out in the《China Child Development Program (2011–2020)》 [3]. In recent years, intervention models based on Internet technology have gradually been applied in the field of breastfeeding research, and successive studies have focused on the impact of Internet intervention models on breastfeeding outcomes. Internet intervention models are defined as digital technology tools and resources used to capture, handle, store, and exchange information via electronic communication [4]. An international board–certified lactation consultant, Heinig, proposed that using the internet to deliver breastfeeding interventions is a way forward to promote breastfeeding [5]. Internet intervention models are support and education programs provided through the internet, aiming to help mothers better understand the importance of breastfeeding and provide techniques and resources to increase the success rate of breastfeeding [6]. These models typically include online courses, interactive discussion forums, email support, video tutorials, mobile applications, etc. Many websites provide breastfeeding educational resources to help mothers understand correct breastfeeding techniques and knowledge, including the benefits of breastfeeding, correct breastfeeding postures and techniques, the frequency and duration of breastfeeding and the common problems and solutions in the breastfeeding process [7]. And social media platforms such as Facebook, Weibo, etc. can help mothers establish online communities to share breastfeeding experiences and problems, and support and encourage each other [8]. In addition, through the online consulting service of the website, mothers can ask questions to experts or experienced breastfeeding consultants and obtain personalized advice and guidance [9]. Some mobile applications, such as breastfeeding trackers, breastfeeding schedules, etc., can help mothers track their babies' eating time and milk volume, and provide personalized feeding suggestions [10].

Studies have shown that pushing relevant knowledge during pregnancy and postpartum through WeChat platforms, mobile phone text messages, and other online education platforms can improve breastfeeding self-confidence, breastfeeding knowledge, and breastfeeding attitudes of puerperas [11, 12]. There is a meta-analysis study on the quantitative analysis of breastfeeding outcomes based on the Internet intervention model [13]. However, this study shows that the Internet intervention model has no effect on improving breastfeeding self-confidence and postpartum short-term exclusive breastfeeding rate. which is controversial compared with the results of recent studies. Therefore, research on the impact of Internet-based intervention models on breastfeeding effects is still worthy of further exploration. Through Meta-analysis, this study clarifies the effect of Internet intervention mode in breastfeeding, and provides evidence-based evidence for promoting the application of Internet intervention mode in breastfeeding.

## Methods

### Search strategy

Systematically searched PubMed, Embase, Web of Science, Cochrane Library, CINAHL, CNKI, Wanfang, CBM and VIP database to collect randomized controlled trials studies on the effect of Internet intervention mode on breastfeeding. The search period is from the establishment of the database to March 2023. The search methods used were Medical Subject Headings (MeSH) combined with free text words. This study was registered in PROSPERO (CRD42023414621) (Additional file 1).

The English search terms were these as follows: “Breastfeeding/Breastfed/Milk Sharing/Sharing, Milk/Breastfeeding, Exclusive/Exclusive Breastfeeding/Breastfed Exclusive/Wet Nursing”” “Internet/Online/network/Website/mobile medical/information communication technology/mobile information technology/Wechat/mobile phone/cell phone/smart phone/mobile app/QQ/Computer-based”; The Chinese search terms were as follows:“母乳喂养/纯母乳喂养/母乳饲养/母乳/纯母乳” “互联网/网络/信息支持/移动信息技术/微信/手机/移动APP/短信/视频/网络支持平台”.

### Inclusion criteria and exclusion criteria

According to the PICO principle and the purpose of this study, the inclusion criteria include: (1) The study was designed as a randomized controlled trial, (2) The study population was over 18 years old, (3) The experimental group carried out the intervention mode of Internet technology, or carried out the intervention method with the help of Internet technology on the basis of the control group; the control group only used conventional health education, (4) Outcome measures included: knowledge of breastfeeding; Breastfeeding attitudes; Breastfeeding confidence; Exclusive breastfeeding rate at discharge; Rate of exclusive breastfeeding at 1 month, 6 weeks, 3 months, 4 months and 6 months postpartum. Exclusion criteria include: (1) Non-Chinese and English literature, (2) Replicated published studies, (3) Full text was not available, (4) Literature of grade C quality.

### Study selection and data extraction

Two investigators independently searched for the search terms. After searching, all studies were imported into EndnoteX9 software to remove duplicates, the title and abstract were read for primary screening, the studies were rescreened and information was extracted according to the inclusion and exclusion criteria. Information was extracted by including first author, publication time, country, age of subjects and sample size, intervention measures of experimental and control groups, intervention time, outcome indicators.

### Quality evaluation of the included literature

Two researchers independently assessed the literatures according to the Cochrane system evaluation manual (5.1.0) [14], including selection bias (sequence generation and allocation concealment); performance bias (blinding of participants and providers), detection bias (blinding of outcome assessors); attrition bias (completeness of outcome data); reporting bias (selective outcome reporting); and other sources of bias. The evaluation grades were divided into three grades, which meet the above standards fully as grade A; partly meet the sets as grade B; completely not meet the sets as grade C. In case of disagreement, a third party was consulted or asked to judge until an agreement was reached.

### Meta-analysis

Meta-analysis was performed using RevMan 5.3. First, a heterogeneity test was performed on the included studies. If *P* ≥ 0.1 and *I*^*2*^ < 50%, it indicated that there was low heterogeneity between the studies, and the fixed effect model was used. If *P* < 0.1 and *I*^*2*^ ≥ 50%, it indicated that there was high heterogeneity between studies. The random-effects model was used to further analyze the source of heterogeneity through sensitivity analysis and subgroup analysis. We reported results as relative effects standardized mean differences (SMD), and risk ratios. Continuous data, we calculated (*SMD*) when similar outcomes were measured using different scales to express the effect size in relation to the variability of the study. Dichotomous data were described by risk ratio (RR), and 95% confidence interval (*CI*) was calculated for each effect.

## Results

### Study selection

**Fig. 1:**
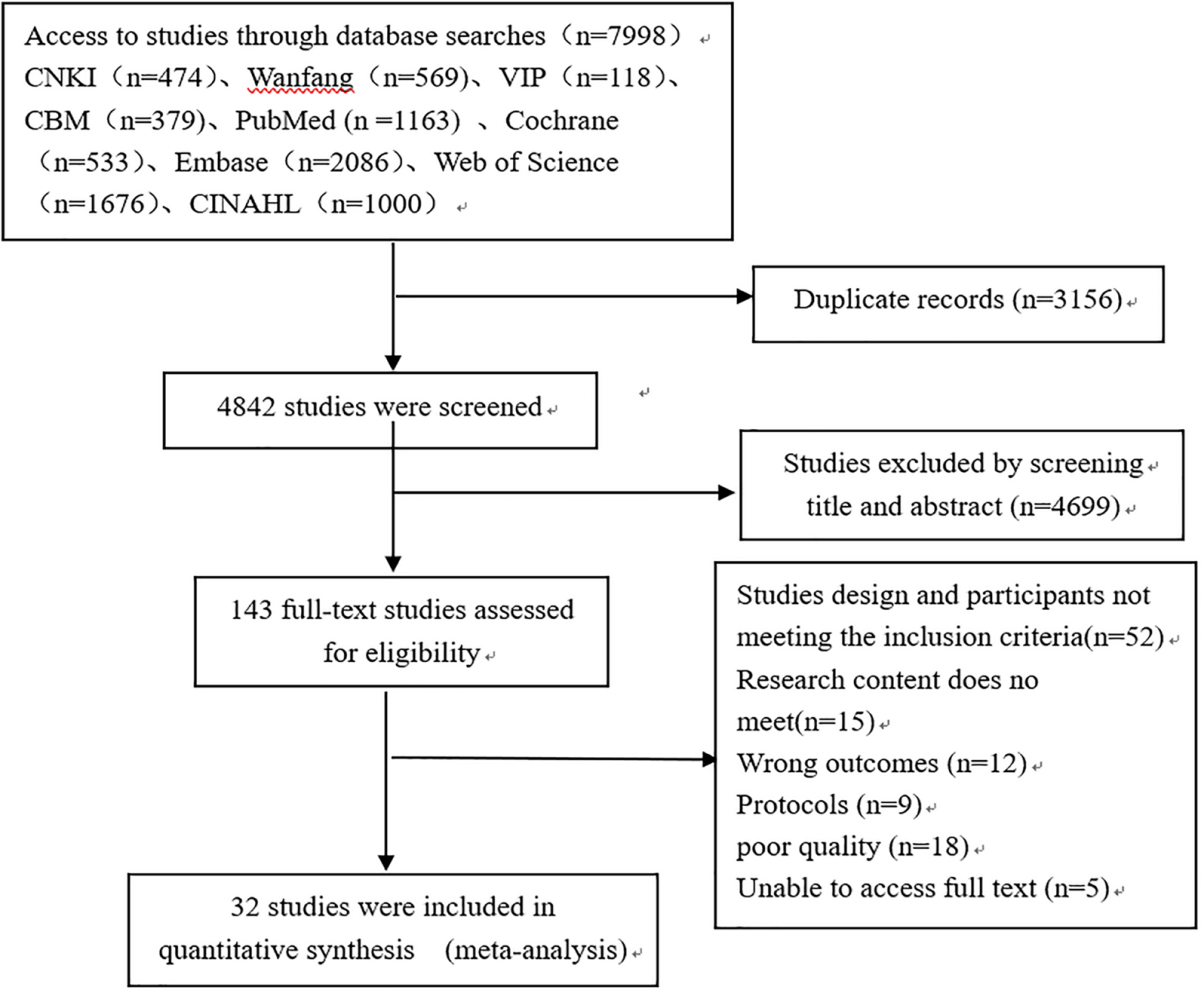
Flow diagram of literature screening

A total of 7998 studies were initially screened using the established search strategy, 3156 duplicates were excluded, 4699 unrelated literatures were excluded by reading titles and abstracts, 143 literatures were obtained after primary screening, and 110 literatures were excluded by reading the full text. Finally, 32 literatures were included. The literature screening process is shown in Fig. 1.

### General information regarding the included literature

A total of 33 articles were included, including 16 English articles [12, 15–29] and 16 Chinese articles [11, 30–44], all of which were RCTs. The combined sample size was 9514 cases, including 4514 cases in the experimental group and 5000 cases in the control group, General information about the 33 studies was shown in Table 1.

**Table 1:** Characteristics of included studies

Author/year (country)	Sample (I/C)	Mean age (I/C)	Intervention time and method	Control	Outcome
Sari 2020 [15] (Turkey)	35/36	I = 27.54 ± 2.71 C = 27.52 ± 3.74	Late pregnancy; web-based neonatal care education and provide breastfeeding knowledge	Usual care	Exclusive breastfeeding rate at 3 months postpartum
Hannula 2014 [16] (Finland)	431/274	I = 30.7 ± 4.1 C = 30.9 ± 4.6	20 weeks gestation to 1 year postpartum; Online Breastfeeding Consultation Platform	Usual care	Breastfeeding attitude; Breastfeeding confidence; Breastfeeding rate at discharge
Seguranyes 2014 [17] (Spain)	276/718	I = 31.22 ± 4.71 C = 31.13 ± 4.76	6 weeks postpartum; Provide CD-ROM and breastfeeding telephone consultation system	Usual care	Breastfeeding rate at discharge;
Galleoas 2014 [18] (Australia)	114/86	I = 31 ± 4.0 C = 30 ± 5.0	Within 8 weeks postpartum; Push breastfeeding knowledge through SMS	Usual care	Exclusive breastfeeding rate at 3 months postpartum
Labarèr 2011 [19] (France)	236/258	No reported	Within 26 weeks postpartum; provide CD-ROM and online consultation platform	Usual care	Exclusive breastfeeding rate at 1 month postpartum
Scott 2013 [20] (USA)	48/50	No reported	6 weeks postpartum; Breastfeeding online health education platform, and SMS push breastfeeding knowledge	Usual care	Breastfeeding attitude
Fahami 2013 [21] (Iran)	36/36	No reported	Within 2 weeks postpartum; Breastfeeding health education APP	Usual care	Breastfeeding knowledge
Bonuck2014 [22] (USA)	129/133	I = 28.2 ± 5.9 C = 28.1 ± 5.6	20 weeks gestation to 3 months postpartum; Postpartum telephone follow-up and home visits to provide breast milk health knowledge	Usual care	Breastfeeding rate at discharge; Exclusive breastfeeding rate at 1 month postpartum; Exclusive breastfeeding rate at 3 months postpartum; Exclusive breastfeeding rate at 6 months postpartum
Ahmed 2016 [23] (USA)	49/57	I = 30.7 ± 4.1 C = 30.9 ± 4.6	From 20 weeks of gestation to 1 year postpartum; Online breastfeeding counseling platform	Usual care	Breastfeeding rate at discharge; Exclusive breastfeeding rate at 1 month postpartum; Exclusive breastfeeding rate at 3 months postpartum;
Seyyedi 2021 [24] (Iran)	40/40	No reported	3 months postpartum; Breastfeeding Health Education APP and mobile phone text messages provide breastfeeding knowledge	Usual care	Breastfeeding attitude; Breastfeeding knowledge; Breastfeeding confidence
Uzunçakmak 2022 [25] (Turkey)	31/37	No reported	6 months postpartum; Breastfeeding Health Education APP provides breastfeeding knowledge	Usual care	Breastfeeding confidence
Hmone 2023 [12] (Australia)	179/174	No reported	From the third trimester to 6 months postpartum; SMS text messages for breastfeeding information	Usual care	Breastfeeding knowledge; Breastfeeding confidence; Exclusive breastfeeding rate at 1 month postpartum; Exclusive breastfeeding rate at 3 months postpartum; Exclusive breastfeeding rate at 4 months postpartum
Patel 2018 [26] (India)	519/518	No reported	From the third trimester to 6 months postpartum; Postpartum telephone follow-up and mobile phone text messages to provide breastfeeding knowledge	Usual care	Exclusive breastfeeding rate at 6 weeks postpartum; Exclusive breastfeeding rate at 3 months postpartum; Exclusive breastfeeding rate at 6 months postpartum
Shen 2013 [42] (China)	211/203	No reported	From 16 weeks of pregnancy to delivery; mobile phone text messages provide breastfeeding knowledge	Usual care	Exclusive breastfeeding rate at 4 months postpartum
Wang 2019 [43] (China)	129/129	I = 27.51 ± 6.19 C = 28.16 ± 6.31	From the third trimester to 6 months postpartum; Postpartum telephone follow-up and WeChat platform provides breastfeeding knowledge	Usual care	Breastfeeding confidence; Exclusive breastfeeding rate at 1 month postpartum; Exclusive breastfeeding rate at 3 months postpartum; Exclusive breastfeeding rate at 6 months postpartum;
Guo 2017 [44] (China)	100/100	I = 31.1 ± 1.2 C = 30.5 ± 3.8	6 weeks postpartum; WeChat platform provides breastfeeding knowledge	Usual care	Breastfeeding knowledge; Exclusive breastfeeding rate at 6 weeks postpartum
Gao 2019 [30] (China)	85/82	I = 23.97 ± 7.28 C = 25.78 ± 6.22	Within 42d postpartum; WeChat platform provides breastfeeding knowledge	Usual care	Exclusive breastfeeding rate at 6 weeks postpartum
Fang 2018 [31] (China)	150/150	I = 23.01 ± 2.66 C = 23.38 ± 2.51	Childbirth to 6 months postpartum; WeChat platform provides breastfeeding knowledge	Usual care	Exclusive breastfeeding rate at 6 months postpartum
Yu 2019 [32] (China)	83/80	I = 28.0 ± 2.6 C = 27.8 ± 2.4	First trimester to 42 days postpartum; WeChat platform provides breastfeeding knowledge	Usual care	Breastfeeding knowledge; Breastfeeding confidence; Breastfeeding rate at discharge; Exclusive breastfeeding rate at 6 weeks postpartum
Wu 2020 [27] (China)	108/109	No reported	12 weeks pregnant to 6 months postpartum; WeChat platform provides breastfeeding knowledge	Usual care	Exclusive breastfeeding rate at 1 months postpartum; Exclusive breastfeeding rate at 3 months postpartum; Exclusive breastfeeding rate at 4 months postpartum
Cai 2016 [33] (China)	460/661	No reported	Discharged to 8 months postpartum; WeChat platform provides breastfeeding knowledge	Usual care	Exclusive breastfeeding rate at 3 months postpartum; Exclusive breastfeeding rate at 6 months postpartum
Ding 2016 [34] (China)	79/79	I = 28.00 ± 2.64 C = 27.76 ± 2.40	From 20 to 28 weeks of gestation to delivery; Mobile phone text messages, QQ or wechat provide breastfeeding knowledge	Usual care	Breastfeeding attitude; Exclusive breastfeeding rate at 1 month postpartum; Exclusive breastfeeding rate at 4 months postpartum; Exclusive breastfeeding rate at 6 months postpartum
Zhang 2018 [35] (China)	50/50	I = 27.56 ± 3. 17 C = 28.02 ± 2. 41	Discharged to 6 months postpartum; WeChat and QQ push breastfeeding knowledge	Usual care	Breastfeeding attitude; Breastfeeding knowledge; Exclusive breastfeeding rate at 1 month postpartum; Exclusive breastfeeding rate at 3 months postpartum; Exclusive breastfeeding rate at 6 months postpartum
Zhang 2019 [36] (China)	61/45	No reported	Within 42d postpartum; WeChat platform provides breastfeeding knowledge	Usual care	Exclusive breastfeeding rate at 6 weeks postpartum;
Zou 2020 [37] (China)	100/100	I = 30.91 ± 3.80 C = 28.01 ± 4.30	Childbirth to 6 months postpartum;	Usual care	Exclusive breastfeeding rate at 6 weeks postpartum; Exclusive breastfeeding rate at 6 months postpartum
Li 2023 [38] (China)	66/65	No reported	From the third trimester to 6 months postpartum; Breastfeeding health education APP and WeChat platform	Usual care	Exclusive breastfeeding rate at 6 months postpartum
Ji 2022 [39] (China)	120/120	I = 23.87 ± 6.58 C = 24.01 ± 6.95	Late pregnancy; Breastfeeding health education APP and WeChat platform	Usual care	Exclusive breastfeeding rate at 6 weeks postpartum;
Jiang 2014 [28] (China)	265/286	No reported	28 weeks gestation to 1 year postpartum; Push breastfeeding knowledge through SMS	Usual care	Exclusive breastfeeding rate at 6 months postpartum; Exclusive breastfeeding rate at 6 months postpartum
Huang 2007 [45] (China)	60/60	I = 29.3 ± 3.1 C = 29.4 ± 3.61	First trimester lasts until 2 weeks postpartum; Online breastfeeding consultation platform and postpartum telephone follow-up	Usual care	Breastfeeding knowledge; Breastfeeding attitude; Breastfeeding rate at discharge; Exclusive breastfeeding rate at 1 month postpartum; Exclusive breastfeeding rate at 6 weeks postpartum
Zhang 2015 [40] (China)	100/100	No reported	Within 42d postpartum; Remote network video breastfeeding health education	Usual care	Exclusive breastfeeding rate at 6 weeks postpartum
Luo 2020 [41] (China)	124/124	No reported	8 weeks of gestation until delivery; Wechat platform provides breastfeeding knowledge	Usual care	Breastfeeding rate at discharge
Zhou 2022 [11] (China)	40/40	No reported	From 24 weeks of pregnancy to 42 days postpartum; Breastfeeding online health education learning platform	Usual care	Exclusive breastfeeding rate at 6 weeks postpartum; Exclusive breastfeeding rate at 4 months postpartum; Exclusive breastfeeding rate at 6 months postpartum

I experimental group; C control group

### Quality evaluation of the included literature

**Fig. 2:**
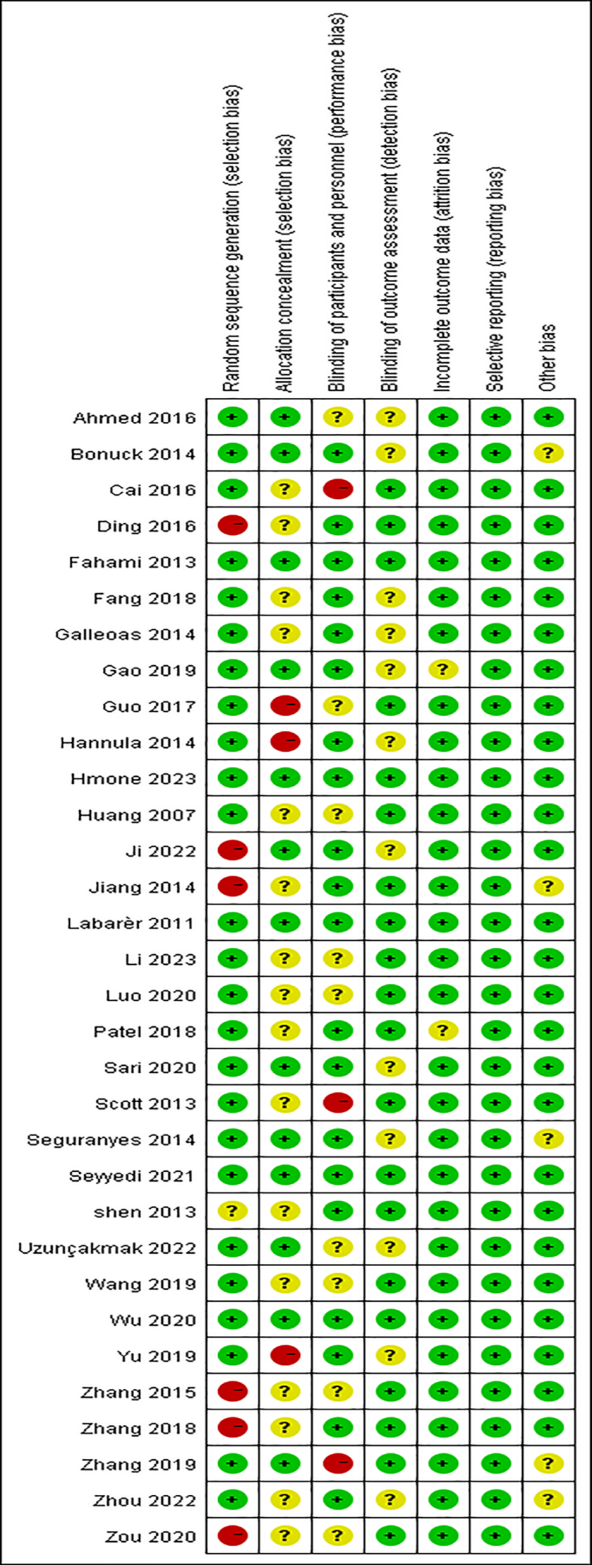
Risk of bias in the included studies

A total of 32 RCTs were included in this study, all of which clearly defined the inclusion and exclusion criteria of subjects, were comparable at baseline, and the measurement results of the experimental and control groups were measured using the same tools and the same statistical methods. All 32 studies [12, 15–29] described the generation method of random sequence, and 16 studies [12, 15–17, 19, 21–25, 27, 30, 32, 36, 39, 44] described allocation hiding; 20 studies [12, 15–19, 21, 22, 24, 26, 28–31, 34, 39] describe blinding the research subjects and intervention implementers, and 20 studies [12, 19–21, 24, 26–28, 33–38, 40–45] describe blinding the outcome evaluators. The quality level of 5 studies is A level, while the rest are B level, and the overall quality was at the medium level or above. The results of the risk of bias assessment were shown in Fig. 2.

### Meta-analysis results

#### Effect of the Internet intervention model on breastfeeding knowledge

**Fig. 3:**
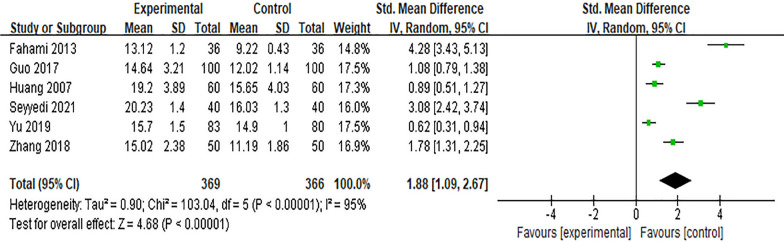
Influence of the Internet intervention model on breastfeeding knowledge

Six studies [14, 17, 22, 25, 28, 37] reported the impact of the Internet intervention model on breastfeeding knowledge. Due to different evaluation tools used, *SMD* was selected for the combination of effect sizes, and the results showed significant heterogeneity (*P* < 0.01, *I*^*2*^ = 95%). After excluding the study of Guo Yuxin^[44]^by sensitivity analysis, there was no significant decrease in heterogeneity, and the random effects model was used for meta-analysis. The results showed that after intervention, the score of breastfeeding knowledge in the experimental group was higher than that in the control group, and the difference was statistically significant [*SMD* = 1.88, 95% *CI* (1.09, 2.67), *P* < 0.01], as shown in Fig. 3.

#### Effect of the Internet intervention model on breastfeeding attitude

**Fig. 4:**
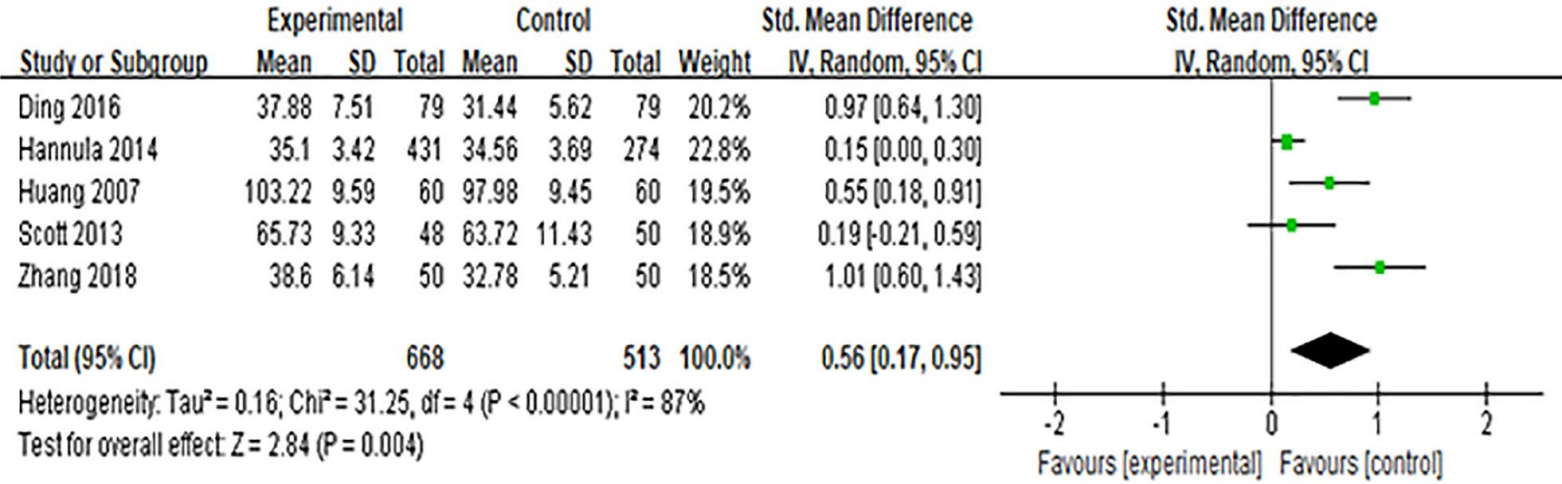
Influence of the Internet intervention model on breastfeeding attitude

Five studies [16, 20, 34, 35, 45] reported the effect of the Internet intervention model on attitudes towards breastfeeding. *SMD* was used to combine effect sizes, and the results showed significant heterogeneity (*P* < 0.01, *I*^*2*^ = 87%). After excluding the study of Hannula [16] by sensitivity analysis, there was no significant decrease in heterogeneity, and the random effects model was used for meta-analysis. The results showed that the scores of breastfeeding attitude in the experimental group were higher than those in the control group after intervention, and the difference was statistically significant [*SMD* = 0.56, 95%*CI* (0.17, 0.95), *P* = 0.004], which was shown in Fig. 4

#### Effect of the Internet intervention model on breastfeeding confidence

**Fig.5:**
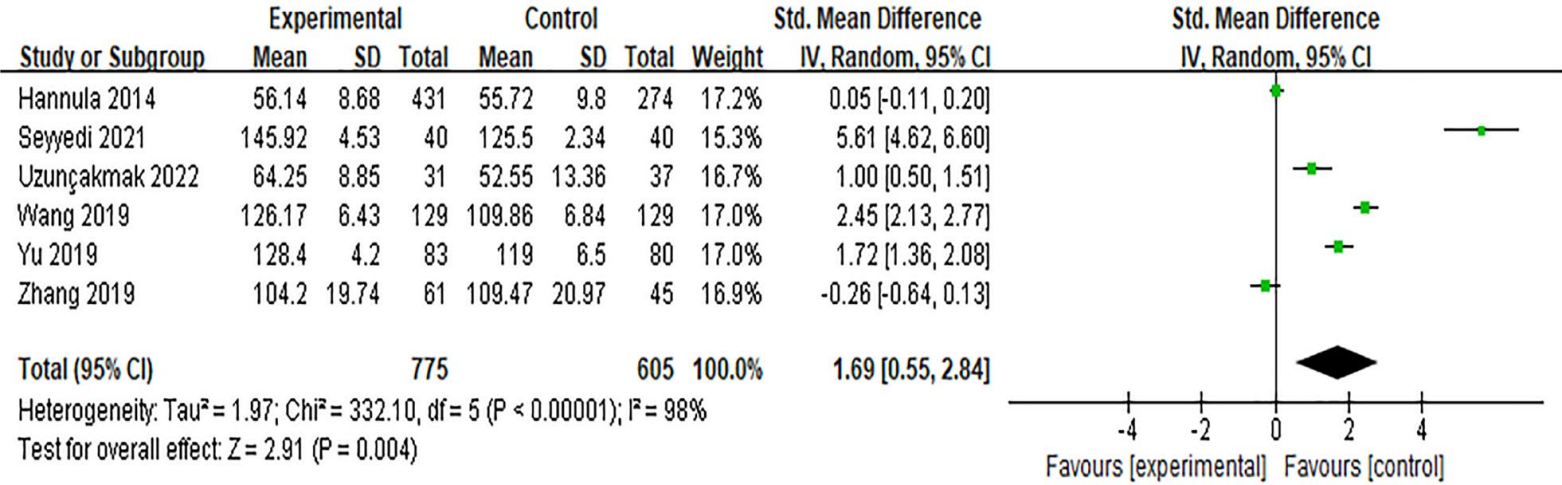
Influence of the Internet intervention model on breastfeeding confidence

Six studies [16, 24, 25, 32, 36, 43] reported the effect of the Internet intervention model on attitudes towards breastfeeding. *SMD* was used to combine effect sizes, and the results showed significant heterogeneity (*P* < 0.01, *I*^*2*^ = 98%). After excluding the study of Hannula [16] by sensitivity analysis, there was no significant decrease in heterogeneity, and the random effects model was used for meta-analysis. The results showed that the scores of breastfeeding confidence in the experimental group were higher than those in the control group after intervention, and the difference was statistically significant [*SMD* = 1.69, 95%*CI* (0.55, 2.84), *P* = 0.004], which was shown in Fig. 5

#### Effect of the Internet intervention model on the rate of exclusive breastfeeding within 6 weeks postpartum

**Fig. 6:**
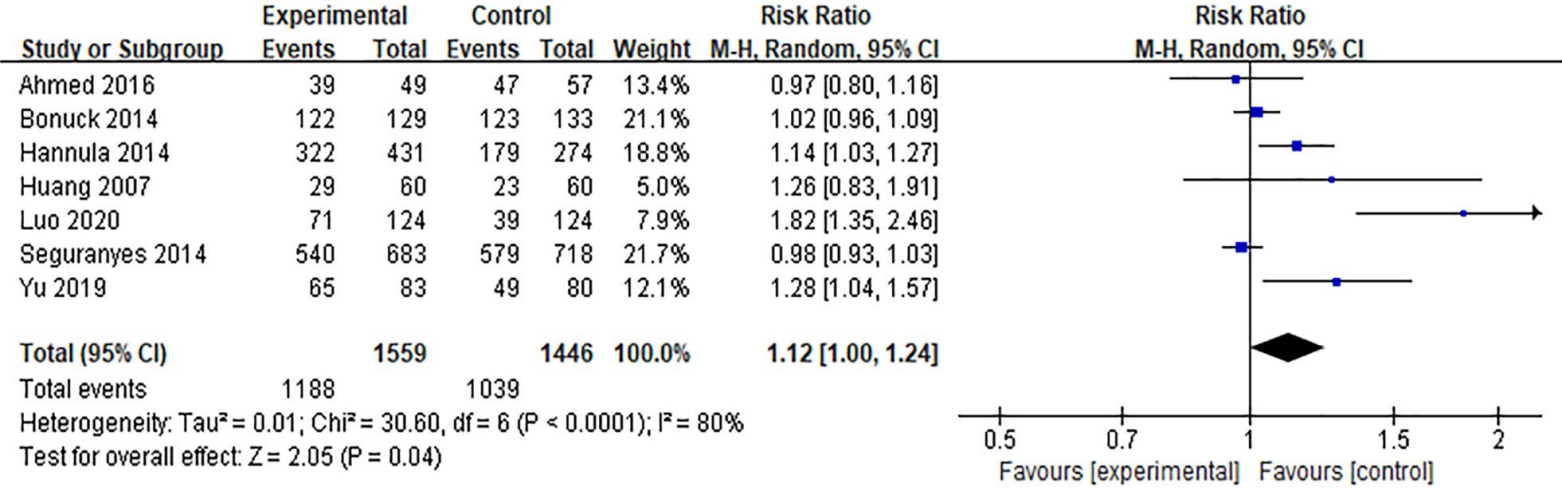
Influence of the Internet intervention model on exclusive breastfeeding at discharge

**Fig. 7:**
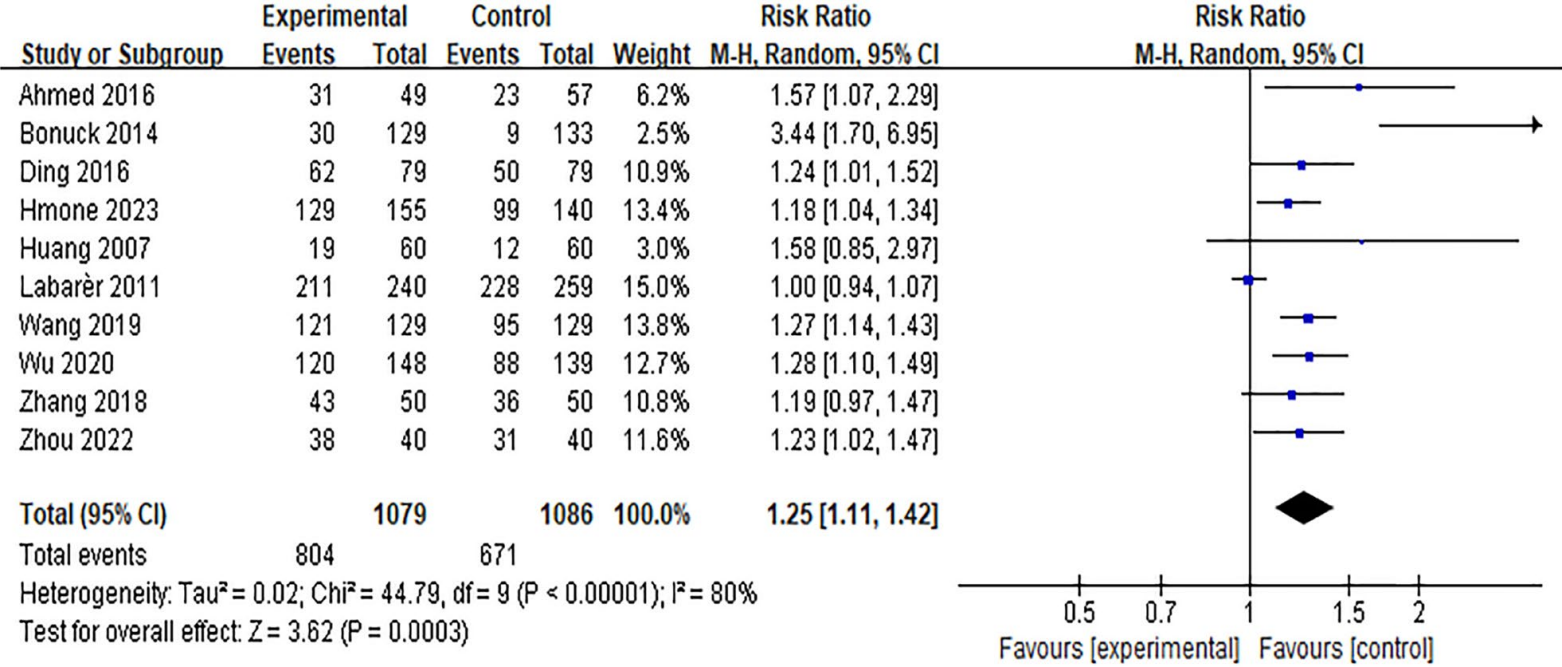
Influence of the Internet intervention model on exclusive breastfeeding at 1 month postpartum

**Fig. 8:**
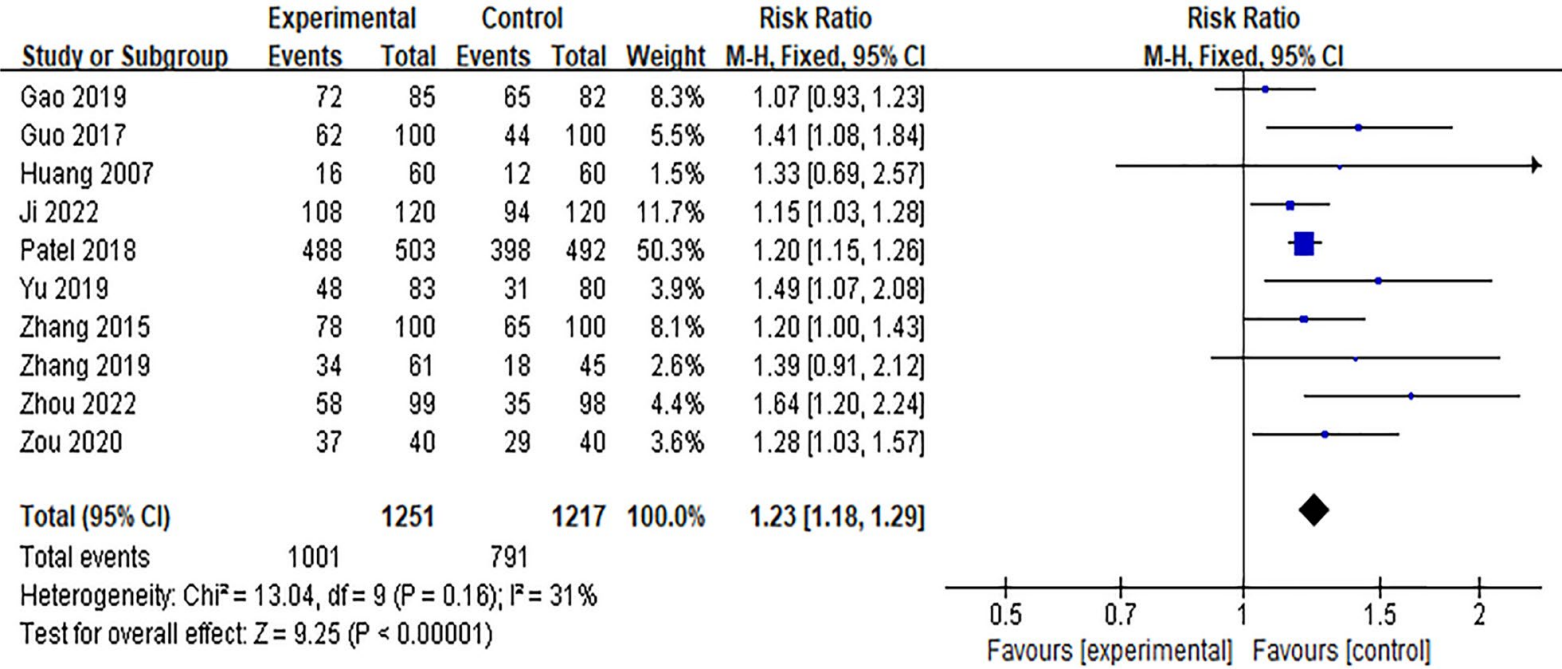
Influence of the Internet intervention model on exclusive breastfeeding at 6 weeks postpartum

Six studies [16, 17, 22, 23, 32, 41, 45] reported the effect of Internet intervention mode on the rate of exclusive breastfeeding at discharge, and the results showed significant heterogeneity (*P* < 0.01, *I*^*2*^ = 80%). After excluding the study of Seguranyes [17] by sensitivity analysis, there was no significant decrease in heterogeneity, and the random effects model was used for meta-analysis. The results showed that the rate of exclusive breastfeeding in the experimental group was higher than that in the control group at discharge after the intervention, and the difference was statistically significant [*RR* = 1.12, 95%*CI* (1.00, 1.24),* P* = 0.04], as shown in Fig. 6. Ten studies [11, 12, 19, 22, 23, 27, 34, 35, 43, 45] reported the effect of Internet intervention model on exclusive breastfeeding rate 1 month postpartum. The results showed that there was significant heterogeneity (*P* < 0.01). The results showed that the exclusive breastfeeding rate at 1 month postpartum in the experimental group was significantly higher than that in the control group [*RR* = 1.25, 95%*CI* (1.11, 1.42), *P* < 0.01], see Fig. 7. Through the sensitivity analysis of these 10 studies, it is found that Labarèr [19] is the main source of heterogeneity. Excluding the study of Labarèr [19], it was found that there was no significant heterogeneity between the studies. Ten studies [11, 26, 30, 32, 36, 37, 39, 40, 44, 45] reported the effect of Internet intervention model on exclusive breastfeeding rate at 6 weeks postpartum, and the results showed no significant heterogeneity. The results showed that the exclusive breastfeeding rate of the experimental group at 6 weeks postpartum was significantly higher than that of the control group [*RR* = 1.16, 95%*CI* (1.13, 1.39), *P* < 0.01], see Fig. 8.

#### Effect of the Internet intervention model on the rate of exclusive breastfeeding at 3 to 6 months postpartum

**Fig. 9:**
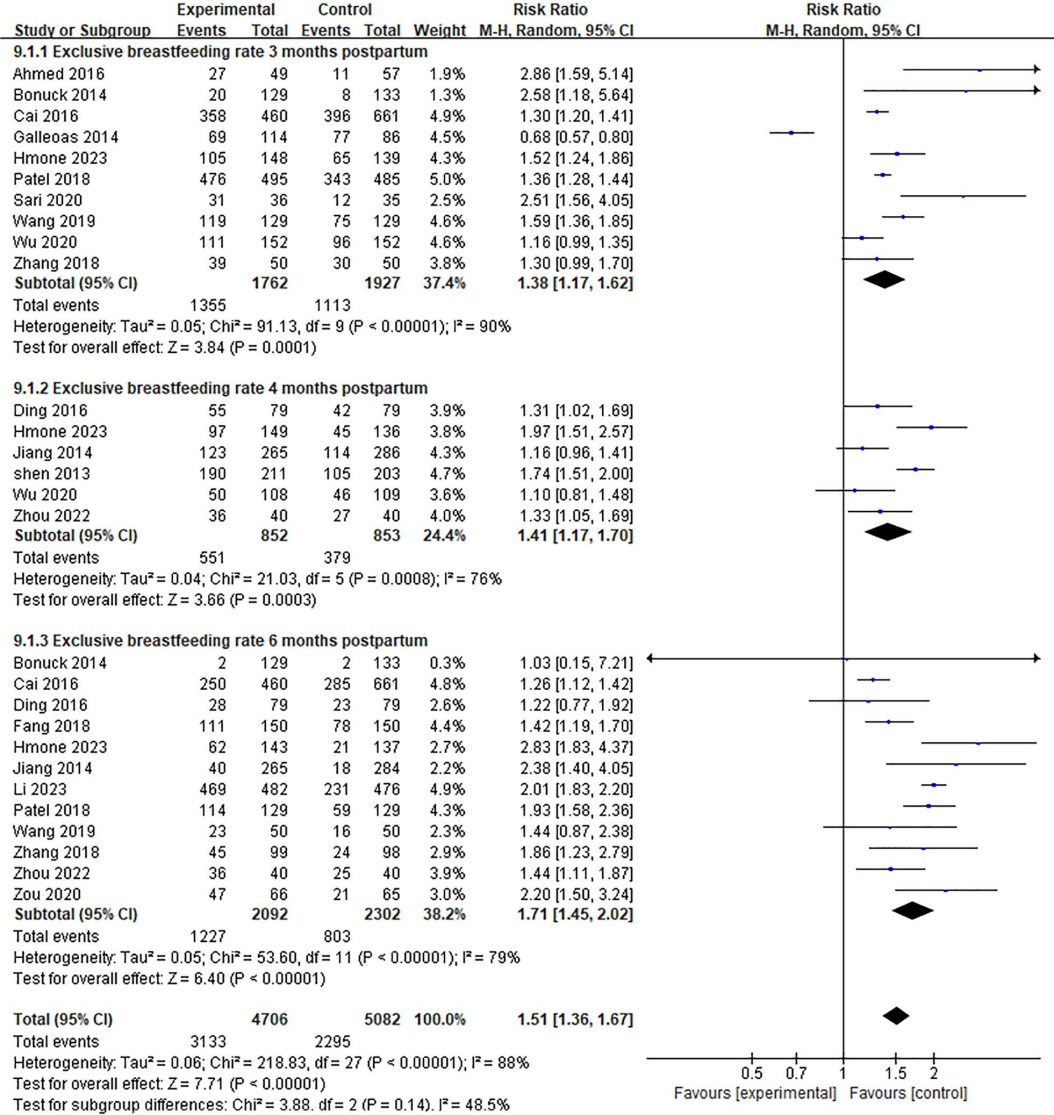
Influence of the Internet intervention model on exclusive breastfeeding at 3–6 months postpartum

Ten studies [12, 15, 18, 22, 23, 26, 27, 33, 43] reported the effect of Internet + intervention model on exclusive breastfeeding rate 3 months Postpartum. The results showed that there was significant heterogeneity (*P* < 0.01). After excluding the study of Cai [33] through sensitivity analysis, the heterogeneity did not decrease significantly, and the random effect model was used for meta-analysis. The results showed that the exclusive breastfeeding rate at 3 months Postpartum in the experimental group was significantly higher than that in the control group [*RR* = 1.38, 95%*CI* (1.17, 1.62), *P* < 0.01], See Fig. 9. Six studies [11, 12, 27, 28, 34, 42] reported the effect of Internet + intervention model on the exclusive breastfeeding rate 4 months studies, and the results were heterogeneous (*P* < 0.01). See Fig. 9. After the study of Shen^[42]^was excluded by sensitivity analysis, the heterogeneity did not decrease significantly, and the random effect model was used for meta-analysis. The results showed that the exclusive breastfeeding rate at 4 months Postpartum in the experimental group was significantly higher than that in the control group [*RR* = 1.41, 95%*CI* (1.17, 1.70), *P* < 0.01], see Fig. 9. Twelve studies [11, 12, 22, 26, 28, 31, 33–35, 37, 38, 43] reported the effect of Internet intervention model on exclusive breastfeeding rate 6 months postpartum. The results showed that there was significant heterogeneity (*P* < 0.01). After excluding the study of Patel [26] by sensitivity analysis, the heterogeneity did not decrease significantly, and the random effect model was used for meta-analysis. The results showed that the exclusive breastfeeding rate at 6 months Postpartum in the experimental group was significantly higher than that in the control group [*RR* = 1.71, 95%*CI* (1.45, 2.02), *P* < 0.01], see Fig. 9.

## Discussion

### Internet intervention model can improve maternal breastfeeding knowledge and attitude, and enhance breastfeeding self-confidence

Meta analysis shows that Internet intervention model can improve the level of maternal breast-feeding knowledge, which is consistent with the results of Lau [8]. The mastery of breastfeeding knowledge is closely related to the effect of breastfeeding, and it is an important condition to promote breastfeeding behavior and improve breastfeeding rate [8]. Some puerperas, especially primipara, lack experience in breastfeeding, while Internet + intervention mode facilitates maternal learning through SMS, Wechat platform or breastfeeding education APP. Puerperas can also learn more about breastfeeding while playing with their mobile phones, which plays an important role in improving their breastfeeding knowledge. Internet intervention model can also improve their attitude towards breastfeeding, which may be related to the intervention lasts from pregnancy to postpartum, and can make uerperas fully aware of the benefits and importance of breastfeeding throughout pregnancy. At the same time, some postpartum women should correctly guide their bad emotions and cognition caused by breast sagging and body deformation after breastfeeding, and instill scientific knowledge. Allay maternal concerns about breastfeeding and improve their breastfeeding attitudes [35]. In addition, this study shows that Interne intervention model also plays a positive role in improving maternal breastfeeding self-confidence, which may be related to the fact that this intervention method can make them exposed to more professional knowledge of breastfeeding and learn feeding skills, and communication with professionals can improve their self-confidence. The results of Du [13] show that the effect of Interne intervention model on improving maternal breast-feeding self-confidence is not statistically significant, which may be related to the fact that only three studies were included and too few subjects were included. In addition, this study analyzes the results of breastfeeding knowledge, attitude and self-confidence, and the results are obviously heterogeneous, which may be caused by the imperfection of random allocation and hiding methods in some studies, and the different duration of intervention methods. Therefore, it is suggested that a more standardized and rigorous intervention program should be designed in the future intervention, and multicenter, high-quality randomized controlled trials should be conducted to further verify the effect.

### The Internet intervention model can increase the rate of exclusive breastfeeding for 6 months postpartum

This study shows that the Internet intervention model can increase the rate of exclusive breastfeeding within 6 months postpartum. The reasons are as follows: initiation time of lactation is closely related to breastfeeding, and has a direct impact on subsequent breastfeeding methods. Most of puerperas with delayed initiation of lactation will have insufficient milk production, and are more likely to give up breastfeeding or add milk powder early [46]. The Internet intervention model lasts from pregnancy to postpartum. Providing sufficient professional knowledge and breastfeeding skills during pregnancy and childbirth can increase maternal lactation cognition and promote early postpartum lactation. These can not only promote the start of lactation, but also help puerpera spends 1 to 2 weeks after delivery, which is a critical period for establishing breastfeeding behavior [38], and increases the rate of exclusive breastfeeding at the time of discharge. In addition, breastfeeding may encounter feeding difficulties, breast pain, blockage and other related problems, which will increase the probability of mothers giving up breastfeeding. It is easier for pregnant women to obtain relevant solutions through WeChat platforms, videos, etc. to promote the implementation of breastfeeding, Increase the rate of exclusive breastfeeding within 6 months postpartum. However, the research results of Du [13]showed that the Internetintervention model had no effect on increasing the rate of exclusive breastfeeding at discharge, within 6 weeks, 3 months, and 4 months postpartum, which may be related to the small number of included literatures, the low quality of some literatures, and the lack of rigorous research design and easy deviation. To sum up, it is suggested that medical staff can build a breastfeeding education network platform throughout pregnancy to postpartum. On the basis of the conventional perinatal education model, a professional-led pregnancy and perinatal education model with mobile phones and the Internet and SMS, wechat and other software as tools should be added, so as to improve the trustability of online education. In the meantime, more well-designed randomized controlled trials are necessary to further explore its effect on the rate of exclusive breastfeeding within 6 months postpartum.

## Limitations

Despite its comprehensive nature, this study also had limitations. First this study only searched Chinese and English literature, and did not search for gray literature. The number of finally included literature is limited, which may have an impact on the comprehensiveness of the results; Second, the quality evaluation grade of most of the included literatures was B, and some literatures had small sample size and poor methodological quality, and did not mention the high heterogeneity of partial merge results caused by allocation hiding and blind methods.

## Conclusion

The results of this study show that the Internet intervention model can improve breastfeeding knowledge, attitude, self-confidence and exclusive breastfeeding rate within 6 months postpartum. The mobile information platform based on Internet provides a good channel for breastfeeding health education. In the process of clinical practice, a more systematic intervention plan should be formulated to improve its effect on breastfeeding. Therefore, in the future, large-sample, multi-center, high-quality randomized controlled studies should be conducted to further explore the specific effects of the Internet intervention model on breastfeeding.

### Supplementary Information

**Additional file 1: Methods.**

## Data Availability

All data generated or analyzed data during this review are included in this article and its Additional file.
