# Supplementary material for: Effect of online intervention mode on breastfeeding results: a systematic review and meta-analysis
Source: Reprod Health. 2023 Nov 6;20:164. doi: 10.1186/s12978-023-01701-0 (PMC10626799; doi:10.1186/s12978-023-01701-0)
Supplement: Supplementary file 1 — Additional file 1: Methods. [file 12978_2023_1701_MOESM1_ESM.docx]

**Additional file 1: Methods**

Search terms used for systematic review:(((((((((Breastfeeding[MeSH Terms])) OR (Breastfed [Title/Abstract])) OR (Milk Sharing[Title/Abstract])) OR (Sharing, Milk[Title/Abstract])) OR ( Breastfeeding, Exclusive[Title/Abstract])) OR (Exclusive Breastfeeding[Title/Abstract])) OR (Breastfed Exclusive[Title/Abstract])) OR (Wet Nursing[Title/Abstract])) AND (((((((((((((((Internet [MeSH Terms)) OR (Online[Title/Abstract])) OR (network[Title/Abstract])) OR (Website[Title/Abstract])) OR (mobile medical[Title/Abstract])) OR ( information communication technology[Title/Abstract])) OR (mobile information technology[Title/Abstract])) OR (Wechat[Title/Abstract])) OR (mobile phone[Title/Abstract])) OR (cell phone[Title/Abstract])) OR (smart phone[Title/Abstract])) OR (mobile app[Title/Abstract])) OR (QQ[Title/Abstract])) OR (Computer-based [Title/Abstract]))

(主题: 母乳喂养(精确)) OR (篇关摘: 纯母乳喂养 (精确) OR (篇关摘:母乳饲养 (精确) ) OR (篇关摘: 母乳 (精确) ) OR (篇关摘: 纯母乳 (精确) ) AND ((主题: 互联网(精确)) OR (篇关: 网络) OR (篇关摘: 信息技术) OR (篇关摘: 移动信息技术) OR (篇关摘: 手机) OR (篇关摘: 微信) OR (篇关摘: 移动APP) OR (篇关摘: 短信) OR (篇关摘: 视频) OR(篇关摘: 网络支持平台) )
